# An exploratory study of knowledge, attitudes, and practices toward HPV associated anal cancer among Pakistani population

**DOI:** 10.3389/fonc.2023.1257401

**Published:** 2023-10-25

**Authors:** Usman Ayub Awan, Wajiha Naeem, Aamer Ali Khattak, Tahir Mahmood, Shehrish Kamran, Suliman Khan, Xingyi Guo, Zhao Yongjing, Jianbo Liu, Abdul Nasir

**Affiliations:** ^1^ Medical Research Center, The Second Affiliated Hospital of Zhengzhou University, Zhengzhou, China; ^2^ Department of Medical Laboratory Technology, The University of Haripur, Haripur, Khyber Pakhtunkhwa, Pakistan; ^3^ School of Computing, Engineering and Physical Sciences, University of the West of Scotland, Paisley, United Kingdom; ^4^ Department of Pathology, Shaukat Khanum Memorial Cancer Hospital and Research Center (SKMCH&RC), Lahore, Pakistan; ^5^ Division of Epidemiology, Department of Medicine, Vanderbilt Epidemiology Center, Vanderbilt-Ingram Cancer Center, Vanderbilt University School of Medicine, Nashville, TN, United States; ^6^ Department of Biomedical Informatics, Vanderbilt University School of Medicine, Nashville, TN, United States; ^7^ Zhengzhou Key Laboratory of Children’s Infection and Immunity , Children’s Hospital Affiliated to Zhengzhou University, Zhengzhou, China; ^8^ Henan Key Laboratory of Precision Diagnosis of Respiratory Infectious Diseases, Zhengzhou Key Laboratory of Precision Diagnosis of Respiratory Infectious Diseases, The Second Affiliated Hospital of Zhengzhou University, Zhengzhou, Henan, China

**Keywords:** human papillomavirus, anal cancer screening, HPV vaccine, anal pap test, Pakistan, public health, immunization, healthcare

## Abstract

**Background:**

Anal cancer, mainly attributed to human papillomavirus (HPV) infection, is rising in prevalence among the general population in Pakistan. This study aimed to examine the knowledge, attitudes, and practices (KAP) towards anal cancer screening and HPV of the general population in Pakistan.

**Method:**

We surveyed anal cancer KAP using social media and snowball sampling from December 2022 to May 2023. The questionnaire had 16 knowledge, 12 attitudes, 6 practice questions, and socio-demographic variables. We applied validity criteria for inclusion and exclusion and used cutoffs ≥50% for each KAP category. We analyzed data in R with Guttman’s λ2 for reliability, did univariate and bivariate analysis, and reported frequencies, percentages, p-values, coefficients, odds ratios, and 95% confidence intervals.

**Results:**

We surveyed 1620 people and discovered low awareness of HPV and anal cancer causes prevention, and screening (11%-24%), high stigma and embarrassment for screening (54%-70%), strong moral beliefs (89%), condom nonuse (91%), and low engagement in health services and programs (9.1%-14%). Knowledge (75.23%, OR = 1.0984, p = 0.05) was shaped by socio-demographic factors, attitude, and practice, with higher education enhancing knowledge (OR = 1.0984, p = 0.05). Attitude (78.45%, OR = 6.6052, p< 0.001) was influenced by socio-demographic factors, practice, and knowledge as well. Younger females, single, unemployed, students, living with more family members, earning more income, and residing in Islamabad had a more positive attitude (ORs from 1.0115 to 6.6052, p< 0.05), while religion did not affect attitude (p = 0.51). Practice (9.16%, OR = 0.1820, p< 0.001) was determined by socio-demographic factors, knowledge, and attitude. Older males, employed teachers, living with more family members, earning less income, and residing in Islamabad had better practice (ORs from 0.1323 to 3.8431, p< 0.05), but marital status and religion did not influence practice (p > 0.05).

**Conclusion:**

Pakistani young adults need more education, awareness, health services, and programs on HPV and anal cancer, as they have low awareness, high stigma, and socio-cultural challenges. In addition, it is recommended for more research and policy initiatives are needed to address socio-cultural factors and increase anal Pap to overcome anal cancer.

## 1 Introduction

Human Papillomavirus (HPV) infection is the most prevalent sexually transmitted infection (STI) worldwide (1, 2) and can cause both benign (anogenital warts) and malignant lesions (penile, anal, and oropharyngeal malignancies) (3). HPV subtypes differ in their genetic sequence and are categorized as low-risk or high-risk based on their oncogenic potential (4–6). Additionally, 14 HPV types are designated as human carcinogenic, including high-risk HPV (HR-HPV; HPV16, 18, 31, 33, 35, 39, 45, 51, 52, 56, 58, 59, and 68) (7, 8). High-risk HPV strains, predominantly 16 and 18, are responsible for almost all cervical cancer cases. A persistent high-risk HPV16 infection is associated with 86 to 100% of anal malignancies and some head and neck cancers (9). High-grade squamous intraepithelial lesions (HSIL), a precancerous condition that can advance to anal cancer, also predominantly involve HPV16 (2, 9, 10). These HPV subtypes are often associated with anogenital cancers but usually do not produce noticeable warts; therefore, HPV infection may remain unnoticed (11, 12).

HPV causes around 48.6% of the annual cases of anal cancer in men (13). However, the rising rates of anal cancer may be associated with the higher number of sexual partners and the more frequent practice of anal intercourse in the past few decades (14, 15). Pakistan is a diverse and complex country where many different ethnic groups live across vast geographic areas. Each group has distinct characteristics and health risks, which may affect their cancer outcomes. However, the country lacks a unified system for collecting and reporting cancer data, which makes it difficult to obtain accurate and reliable estimates of cancer incidence and prevalence. The existing research on cancer in Pakistan is often based on regional data, which may not reflect the true picture of the whole country or account for the variations among different ethnic groups, and the available data is only hospital-based (16). According to International Agency for Research on Cancer (IARC), in 2020, Pakistan had crude incidence rates of anal cancer of 0.34 and 0.17 per 100,000 for men and women, while age-standardized incidence rates of 0.46 and 0.22 per 100,000 for men and women, respectively (17).

Pakistan is a country where Islam shapes society’s moral and social fabric. Any sexual activity outside the marital bond, especially homosexual sex, is considered a grave sin and a violation of Islamic law. Many Pakistanis also assume that HIV cannot affect them, as they believe Muslims are immune to this disease associated with illicit sex (18, 19). However, this assumption is challenged by the rising number of HIV cases in Pakistan, which reached 8,262 from January to September 2022 (20). The main mode of HIV transmission in Pakistan is sexual contact, especially among men who have sex with men (MSM). MSM in Pakistan includes various groups, such as seamen, prisoners, drug addicts, truck drivers, migrant men, male prostitutes, hijras (transvestites), zenanas (she-males), maalishias (masseurs), and chavas (men who swap sexual roles) (21–24). These groups are highly vulnerable to HIV infection due to their risky sexual behaviors and lack of access to prevention and treatment services. The current HIV epidemic in Pakistan is a wake-up call for the public and the authorities to recognize and address the hidden reality of MSM and their sexual health needs.

Given the increasing burden of sexually transmitted diseases in Pakistan, it is imperative to investigate the basic knowledge and awareness of the population about anal cancer. However, there is a dearth of research on this topic in Pakistan, and the level of awareness and prevention of anal cancer among the public is unknown. Therefore, this study is the first of its kind in Pakistan, aiming to explore the knowledge, attitudes, and practices (KAP) regarding HPV-associated anal cancer in the community.

## 2 Methods

### 2.1 Study design

**Figure 1:**
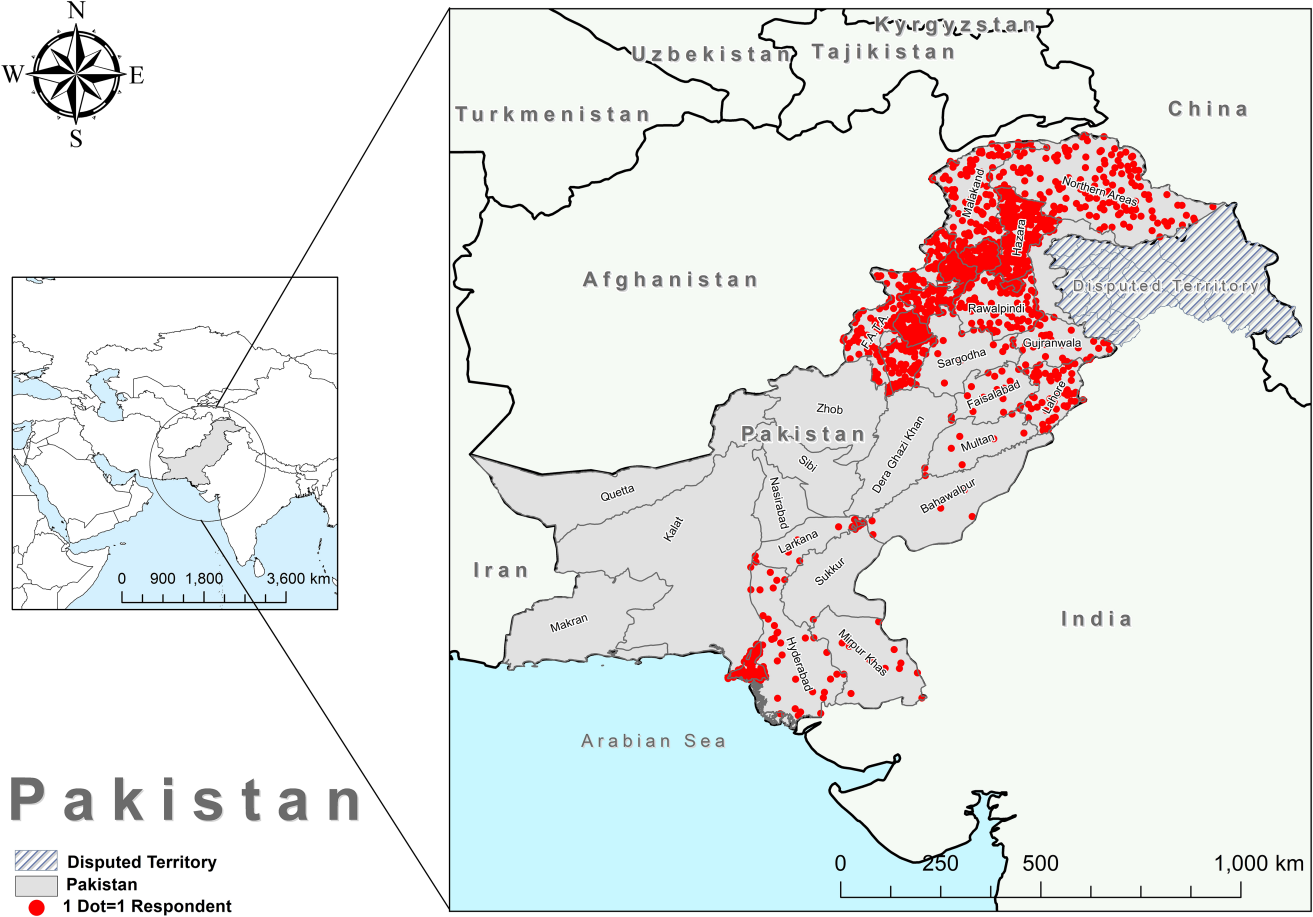
Survey respondents’ distribution and participation numbers in different areas of Pakistan.

We used an anonymous online questionnaire to collect the data for this study, which evaluated the public’s knowledge, attitudes, and practices concerning anal cancer. We employed a snowball sampling technique to distribute the questionnaire link through various social media platforms, such as WhatsApp, Twitter, Facebook, Messenger, Gmail, and Instagram. The respondents had to answer every item on the questionnaire, which was available in both English and their native language. The data collection period for this study spanned seven months, from December 2022 to May 2023. The participants completed the questionnaire voluntarily after receiving information about the nature of the study and assurance of anonymity and confidentiality of responses. The study encompassed all the regions of Pakistan, including Khyber Pakhtunkhwa (KPK), Punjab, Balochistan, Sindh, Gilgit-Baltistan (GB), Azad Jammu Kashmir (AJK), and Islamabad Capital Territory (ICT), as illustrated in **Figure 1**.

### 2.2 Inclusion and exclusion criteria

This study aimed to assess the Pakistani population’s KAP parameters on a large scale, regardless of gender and residence. The study included respondents who met the following criteria: (i) they were 10 years old or above, and (ii) they were residents of Pakistan. These criteria were consistent throughout the study. To ensure the validity and relevance of our study, we excluded respondents who met any of the following criteria: (i) had a history of allergy to the HPV vaccine and could not receive immunization, (ii) the respondent was a foreign citizen studying or living in Pakistan, and (iii) had HPV-associated malignancy, as their personal experience might bias opinions.

### 2.3 Measurements

The questionnaire was developed by adapting previously validated questionnaires to the study’s context (25–31). We measured the respondents’ knowledge of HPV using 16 questions encompassing various topics such as clinical features of anal cancer, viral structure, transmission modes, anal cancer prevention, anal screening, treatment options, and vaccination information. All respondents could opt for “Yes,” “No,” or “Not Sure.” The knowledge scores were derived by allocating one point to each correct answer, resulting in a total score ranging from 0 to 16. A cutoff of ≥50% (eight) was applied to distinguish knowledgeable and not-knowledgeable respondents, with higher scores indicating better knowledge.

Furthermore, we investigated the attitudes toward HPV using 12 questions about individual beliefs, perceptions about anal screening, and willingness toward HPV vaccination. The responses were categorized as “Yes,” “No,” and “Not Sure.” Likewise, the overall attitude was divided into positive and negative scales. The attitude score was obtained by giving one point to “Yes” as the positive attitude and zero points to “No” and “Not Sure” as the negative attitude. A cutoff of six was applied to differentiate positive and negative attitudes, with equal or higher scores indicating a positive attitude and lower indicating a negative attitude.

Additionally, we appraised the practices of participants using 6 questions about screening for anal cancer, participation in HPV awareness campaigns, and safe sex practices. Respondents were asked to answer using the “Yes” and “No” scales, and one point was awarded for good practices. A cutoff of six was applied to classify “good practice” and “poor/bad practices”, where scores equal or above the cutoff indicated good practice while low scores indicated poor/bad practices. Finally, we collected a broad range of socio-demographic variables, including gender, age, marital status, employment status, occupation, number of family members, level of education, religion, monthly household income, and place of residence to categorize their knowledge, attitudes, and practices in different variables, as illustrated in **Table 1**.

**Table 1:** Demographics of participants (N = 1620).

Variables		Frequency (n= 1620)	Percent (%)
Age (years)	10-20	532	32.8
	21-30	872	53.8
	31-40	196	12.1
	41-50	12	0.7
	>50	8	0.5
Gender	Male	628	38.8
	Female	992	61.2
Marital Status	Single	1304	80.5
	Married	300	18.5
	Widowed	4	0.2
	Divorced	12	0.7
Employment	Employed	476	29.4
	Unemployed	1144	70.6
Occupation	Business	44	2.7
	Other	132	8.1
	Employed	144	8.9
	Teacher	200	12.3
	Student	1100	67.9
Family Members	≤ 2	108	6.7
	3	340	21.0
	≥ 4	1172	72.3
Income	≤50,000	248	15.3
	51,000-80,000	188	11.6
	>80,000	220	13.6
	Not disclosed	964	59.5
Education	Primary school	16	1.0
	High school	520	32.1
	University	1084	66.9
Religion	Non-Muslim	12	0.7
	Muslim	1608	99.3
Resident (Province)	ICT	134	8.3
	AJK/GB	146	9.0
	Sindh	149	9.2
	Punjab	223	13.8
	KPK	968	59.8

### 2.4 Ethical approval

Ethical approval was obtained from the Departmental Bioethical Committee of The University of Haripur (UOH-RC-EA-142). Informed consent was taken from all participants before they participated in the study. In the case of participants under 18 years old, the participant’s legal guardian provided written informed consent to participate in this study.

### 2.5 Statistical analysis

We stored the raw data in an Excel file and imported it into R version 4.2.2 for analysis. We used different R packages (gtsummary, ggplot2, and circlize) to clean and screen the data and to estimate the reliability of the test items; in addition, we adopted Guttman’s *λ*_2_ (32) instead of Cronbach’s alpha. Guttman’s *λ*_2_ was used to estimate the reliability of the questionnaire, as it does not require the assumptions of Cronbach’s alpha, such as uncorrelated errors, identical covariances between items, and uni-dimensionality, which are often violated. We obtained a Guttman’s *λ*_2_ value of 0.86, indicating that 86% of the variance was due to true scores and 14% was due to error. Univariate descriptive analysis was performed to summarize the frequency (n) and percentages (%) for each category of the variables under consideration (see **Tables 1**–**4**). We also conducted bivariate descriptive analysis to examine the relationship between socio-demographic and main variables (i.e., knowledge, attitudes, and practices). We reported the frequency (n) and percentages (%) for each sub-category among both variables. Furthermore, the Chi-square test was used to check the association between variables and reported the results in terms of the p-value, where a p-value< 0.05 indicated a significant association between the responses of considered variables. Finally, we attempted binary logistic regression, and interpreted the model using regression coefficient values, the p-value of coefficients, odds ratio (OR), and 95% confidence interval (CI) (see **Tables 5**–**7**).

**Table 2:** Knowledge questionnaire responses.

Questions	Response		
	No	Not sure	Yes
Anal cancer is more common in men than in women?	40 (2.5%)	252 (16%)	1,328 (82%)
Can HPV cause penile cancer in males?	116 (7.2%)	584 (36%)	920 (57%)
Can medications cure HPV infections?	188 (12%)	928 (57%)	504 (31%)
Do you think the HPV vaccine is available in Pakistan?	68 (4.2%)	544 (34%)	1,008 (62%)
Does sexual contact transmit HPV?	172 (11%)	760 (47%)	688 (42%)
Have sex at a young age a greater risk factor for anal cancer?	580 (36%)	868 (54%)	172 (11%)
Have you heard about anal Pap?	360 (22%)	988 (61%)	272 (17%)
Have you heard of the HPV vaccine?	84 (5.2%)	904 (56%)	632 (39%)
HPV cause life-threatening complications?	132 (8.1%)	924 (57%)	564 (35%)
Is HPV a viral disease?	116 (7.2%)	576 (36%)	928 (57%)
Is HPV associated with other cancers other than anal?	848 (52%)	216 (13%)	556 (34%)
Is HPV associated with skin warts?	44 (2.7%)	428 (26%)	1,148 (71%)
People with HPV are at higher risk for anal cancer	112 (6.9%)	1,232 (76%)	276 (17%)
Smoking tobacco is a risk factor for anal cancer?	100 (6.2%)	1,260 (78%)	260 (16%)
Some types of HPV cause genital and anal warts?	116 (7.2%)	476 (29%)	1,028 (63%)
Will using condoms prevent you from getting HPV?	64 (4.0%)	388 (24%)	1,168 (72%)
** Total**	**3,140 (12%)**	**11,328 (44%)**	**11,452 (44%)**

**Table 3:** Attitude questionnaire responses.

Questions	Response		
	No	Not Sure	Yes
Are you well-informed about the diagnosis and treatment of anal cancer	44 (2.7%)	488 (30%)	1,088 (67%)
Asking for a test or getting it might be embarrassing	76 (4.7%)	412 (25%)	1,132 (70%)
How familiar are you with the signs and symptoms of anal cancer?	120 (7.4%)	912 (56%)	588 (36%)
I prefer an anal Pap if the physician is the same gender as me	216 (13%)	532 (33%)	872 (54%)
I think adolescents should be encouraged to get HPV immunization.	104 (6.4%)	444 (27%)	1,072 (66%)
I think HPV vaccination should be mandatory.	972 (60%)	416 (26%)	232 (14%)
I will go for anal Pap if I can collect a sample myself.	80 (4.9%)	384 (24%)	1,156 (71%)
I would like to learn more about the anal pap test	60 (3.7%)	200 (12%)	1,360 (84%)
Multiple sex partners make an individual more likely to get anal cancer?	52 (3.2%)	536 (33%)	1,032 (64%)
Myths or religious beliefs prevent me from HPV vaccination?	60 (3.7%)	404 (25%)	1,156 (71%)
Does social pressure/stigmatization prevent me from getting anal Pap?	48 (3.0%)	480 (30%)	1,092 (67%)
Would you not recommend your family members for anal Pap?	40 (2.5%)	340 (21%)	1,240 (77%)
** Total**	**2,528 (13%)**	**5,548 (29%)**	**11,364 (58%)**

**Table 4:** Practice questionnaire responses.

Questions	Response	
	No	Yes
Do you ever screen yourself for anal cancer?	1,412 (87%)	208 (13%)
Do you practice moral beliefs?	1,436 (89%)	184 (11%)
Do you prefer to use condoms for safe sex?	1,444 (89%)	176 (11%)
Have you ever been screened for anal cancer?	232 (14%)	1,388 (86%)
Have you ever participated in a health education program related to HPV?	1,464 (90%)	156 (9.6%)
Willing to go for anal Pap if I feel any genital warts?	148 (9.1%)	1,472 (91%)
** Total**	**6,136 (63%)**	**3,584 (37%)**

**Table 5:** Knowledge towards HPV by demographic variables.

Variables		Knowledge Grading					Binary Logistic Regression				
		Knowledgeable		Not Knowledgeable		Chi-square P-value	Coefficient	p-value	Odds ratio	95% Confidence Interval	
		N	%	N	%					Lower	Upper
Age	10-20	284	35.50	248	30.24	0.0039	Ref				
	21-30	416	52.00	456	55.61		-0.3537	0.1060	0.7021	0.4561	1.0768
	31-40	88	11.00	108	13.17		-0.5360	0.1130	0.5851	0.3010	1.1348
	41-50	4	0.50	8	0.98		-1.0474	0.2232	0.3508	0.0681	2.0092
	>50	8	1.00	0	0.00		-18.9554	0.9706	0.0000	NA	NA
Gender	Male	309	38.63	319	38.90	0.9493	-0.1521	0.2308	0.8589	0.6692	1.1010
	Female	491	61.38	501	62.63		Ref				
Marital Status	Single	656	82.00	648	79.02	0.0909	-0.0915	0.8990	0.9126	0.2065	3.7161
	Married	140	17.50	160	19.51		0.0475	0.9471	1.0486	0.2394	4.2163
	Widowed	0	0.00	4	0.49		15.9676	0.9825	8602877	9.14E+234	2.76E+251
	Divorced	4	0.50	8	0.98		Ref				
Employment	Employed	216	27.00	260	31.71	0.0429	Ref				
	Unemployed	584	73.00	560	68.29		0.0953	0.6919	1.1000	0.6884	1.7694
Occupation	Business	16	2.00	28	3.41	0.0014	Ref				
	Other	56	7.00	76	9.27		-0.4138	0.3683	0.6612	0.2666	1.6260
	Employed	68	8.50	76	9.27		-0.2901	0.4978	0.7482	0.3211	1.7304
	Teacher	80	10.00	120	14.63		-0.9840	0.0205	0.3738	0.1612	0.8568
	Student	580	72.50	520	63.41		-0.9746	0.0374	0.3773	0.1494	0.9410
Family Members	≤2	40	5.00	68	8.29	0.0032	0.1330	0.6390	1.1422	0.6545	1.9923
	3	188	23.50	152	18.54		Ref				
	≥4	572	71.50	600	73.17		0.4155	0.0052	1.5152	1.1338	2.0328
Income	≤50,000	84	10.50	164	20.00	0.0000	0.7692	0.0019	2.1580	1.3329	3.5188
	51,000-80,000	92	11.50	96	11.71		Ref				
	>80,000	128	16.00	92	11.22		-0.0281	0.9037	0.9723	0.6164	1.5351
	Not disclosed	496	62.00	468	57.07		0.0466	0.8180	1.0477	0.7051	1.5622
Education	Primary school	8	1.00	8	0.98	0.0461	-0.1984	0.7382	0.8201	0.2424	2.5817
	High school	280	35.00	240	29.27		Ref				
	University	512	64.00	572	69.76		0.0938	0.6605	1.0984	0.7226	1.6716
Religion	Non-Muslim	8	1.00	4	0.49	0.3616	-1.5827	0.0390	0.2054	0.0419	0.8584
	Muslim	792	99.00	816	99.51		Ref				
Resident (Province)	ICT	65	8.13	69	8.41	0.8702	0.0804	0.7684	1.0837	0.6345	1.8530
	AJK/GB	73	9.13	73	8.90		Ref				
	Sindh	71	8.88	78	9.51		-0.0147	0.9560	0.9854	0.5834	1.6640
	Punjab	104	13.00	119	14.51		-0.1504	0.5482	0.8604	0.5265	1.4066
	KPK	487	60.88	481	58.66		-0.1342	0.5102	0.8744	0.5873	1.3073
Attitude	Negative	32	4.00	316	38.54	0.0000	Ref				
	Positive	768	96.00	504	61.46		-3.1273	0.0000	0.0438	0.0270	0.0681
Practice	Good	104	13.00	44	5.37	0.0000	Ref				
	Poor	696	87.00	776	94.63		0.9174	0.0000	2.5027	1.6214	3.9293

**Table 6:** Practices towards HPV by demographic variables.

Variables		Attitude Grading					Binary Logistic Regression				
		Negative		Positive		Chi-square P-value	Coefficient	p-value	Odds ratio	95% Confidence Interval	
		N	%	N	%					Lower	Upper
Age	1020	52	14.94	480	37.74	0.0000	Ref				
	21-30	232	66.67	640	50.31		-1.5470	0.0000	0.2129	0.1152	0.3885
	31-40	48	13.79	148	11.64		-1.7254	0.0003	0.1781	0.0702	0.4461
	41-50	8	2.30	4	0.31		-2.8312	0.0010	0.0589	0.0102	0.3069
	>50	8	2.30	0	0.00						
Gender	Male	170	48.85	458	36.01	0.0000	-0.2714	0.1296	0.7623	0.5367	1.0837
	Female	178	51.15	814	63.99		Ref				
Marital Status	Single	264	75.86	1040	81.76	0.0449	0.3211	0.6772	1.3786	0.2779	5.9927
	Married	80	22.99	220	17.30		1.0081	0.1984	2.7404	0.5456	12.3250
	Widowed	0	0.00	4	0.31						
	Divorced	4	1.15	8	0.63		Ref				
Employment	Employed	124	35.63	352	27.67	0.0048	Ref				
	Unemployed	224	64.37	920	72.33		0.5095	0.1183	1.6644	0.8685	3.1235
Occupation	Business	12	3.45	32	2.52	0.0000	Ref				
	Other	44	12.64	88	6.92		0.9313	0.0957	2.5379	0.8333	7.5108
	Employed	16	4.60	128	10.06		2.6582	0.0000	14.2704	4.3751	46.8278
	Teacher	88	25.29	112	8.81		0.2046	0.6731	1.2271	0.4603	3.1115
	Student	188	54.02	912	71.70		1.0363	0.0569	2.8186	0.9474	8.0406
Family Members	≤2	44	12.64	64	5.03	0.0000	-0.1299	0.7087	0.8782	0.4451	1.7421
	3	64	18.39	276	21.70		Ref				
	≥4	240	68.97	932	73.27		0.5398	0.0189	1.7157	1.0889	2.6860
Income	≤50,000	88	25.29	160	12.58	0.0000	-0.1889	0.5186	0.8279	0.4645	1.4647
	51,000-80,000	52	14.94	136	10.69		Ref				
	>80,000	16	4.60	204	16.04		1.8879	0.0000	6.6052	3.1617	14.4197
	Not disclosed	192	55.17	772	60.69		0.2008	0.4268	1.2224	0.7402	1.9963
Education	Primary school	4	1.15	12	0.94	0.0000	-1.3619	0.0736	0.2562	0.0623	1.2494
	High school	56	16.09	464	36.48		Ref				
	University	288	82.76	796	62.58		0.2107	0.4840	1.2346	0.6844	2.2295
Religion	Non-Muslim	4	1.15	8	0.63	0.5153	-1.0937	0.3022	0.3350	0.0492	2.5599
	Muslim	344	98.85	1264	99.37		Ref				
Resident (Province)	ICT	17	4.89	117	9.20	0.0276	0.0115	0.9793	1.0115	0.4274	2.4381
	AJK/GB	27	7.76	119	9.36		Ref				
	Sindh	32	9.20	117	9.20		-0.0726	0.8530	0.9300	0.4308	2.0060
	Punjab	60	17.24	163	12.81		-0.3137	0.3719	0.7307	0.3644	1.4481
	KP	212	60.92	756	59.43		-0.3092	0.3036	0.7340	0.4017	1.3075
Knowledge	Knowledgeable	97	27.87	1118	87.89	0.0000	Ref				
	Not Knowledgeable	251	72.13	154	12.11		-3.1805	0.0000	0.0416	0.0286	0.0594
Practice	Good	12	3.45	136	10.69	0.0001	Ref				
	Poor	336	96.55	1136	89.31		-1.1007	0.0026	0.3326	0.1561	0.6608

**Table 7:** Attitudes towards HPV by demographic variables.

Variables		Practice Grading					Binary Logistic Regression				
		Good		Poor		Chi-square P-value	Coefficient	p-value	Odds ratio	95% Confidence Interval	
		N	%	N	%					Lower	Upper
Age	10-20	24	16.22	508	34.51	0.0000	Ref				
	21-30	96	64.86	776	52.72		-0.5978	0.1467	0.5500	0.2426	1.2187
	31-40	28	18.92	168	11.41		-2.0230	0.0005	0.1323	0.0415	0.4081
	41-50	0	0.00	12	0.82						
	>50	0	0.00	8	0.54						
Gender	Male	92	62.16	536	36.41	0.0000	-1.0236	0.0000	0.3593	0.2374	0.5394
	Female	56	37.84	936	63.59		Ref				
Marital Status	Single	120	81.08	1184	80.43	0.6527	-15.0656	0.9884	0.0000	0.0000	0.0000
	Married	28	18.92	272	18.48		-14.0513	0.9892	0.0000	0.0000	0.0000
	Widowed	0	0.00	4	0.27						
	Divorced	0	0.00	12	0.82		Ref				
Employment	Employed	72	48.65	404	27.45	0.0000	Ref				
	Unemployed	76	51.35	1068	72.55		1.3463	0.0000	3.8431	2.0515	7.1113
Occupation	Business	4	2.70	40	2.72	0.0000	Ref				
	Other	4	2.70	128	8.70		0.4289	0.6029	1.5356	0.2949	8.0462
	Employed	12	8.11	132	8.97		0.7834	0.2578	2.1890	0.5111	8.1261
	Teacher	36	24.32	164	11.14		-0.4780	0.4387	0.6200	0.1622	1.9222
	Student	92	62.16	1008	68.48		-1.0843	0.1110	0.3381	0.0795	1.1964
Family Members	≤2	4	2.70	104	7.07	0.0000	-0.2002	0.7602	0.8186	0.2422	3.3357
	3	12	8.11	328	22.28		Ref				
	≥4	132	89.19	1040	70.65		-1.4768	0.0000	0.2284	0.1126	0.4260
Income	≤50,000	56	37.84	192	13.04	0.0000	-1.7036	0.0000	0.1820	0.0905	0.3498
	51,000-80,000	16	10.81	172	11.68		Ref				
	>80,000	24	16.22	196	13.32		-0.4822	0.1997	0.6174	0.2910	1.2801
	Not disclosed	52	35.14	912	61.96		-0.0074	0.9826	0.9926	0.4985	1.8988
Education	Primary school	0	0.00	16	1.09	0.0000					
	High school	20	13.51	500	33.97		Ref				
	University	128	86.49	956	64.95		-0.3968	0.3477	0.6724	0.2907	1.5222
Religion	Non-Muslim	0	0.00	12	0.82	0.5487					
	Muslim	148	100.00	1460	99.18		Ref				
Resident (Province)	ICT	4	2.70	130	8.83	0.0394	1.2588	0.0400	3.5210	1.1490	13.3843
	AJK/GB	17	11.49	129	8.76		Ref				
	Sindh	18	12.16	131	8.90		0.3045	0.4594	1.3560	0.6036	3.0543
	Punjab	16	10.81	207	14.06		0.7660	0.0669	2.1513	0.9480	4.9224
	KP	93	62.84	875	59.44		0.0583	0.8541	1.0601	0.5550	1.9348
Knowledge	Knowledgeable	136	91.89	1079	73.30	0.0000	Ref				
	Not Knowledgeable	12	8.11	393	26.70		1.4566	0.0002	4.2914	2.0681	9.6165
Attitude	Negative	12	8.11	336	22.83	0.0001	Ref				
	Positive	136	91.89	1136	77.17		-1.1469	0.0039	0.3176	0.1401	0.6699

## 3 Results

### 3.1 Socio-demographic characteristics of study participants

**Figure 2:**
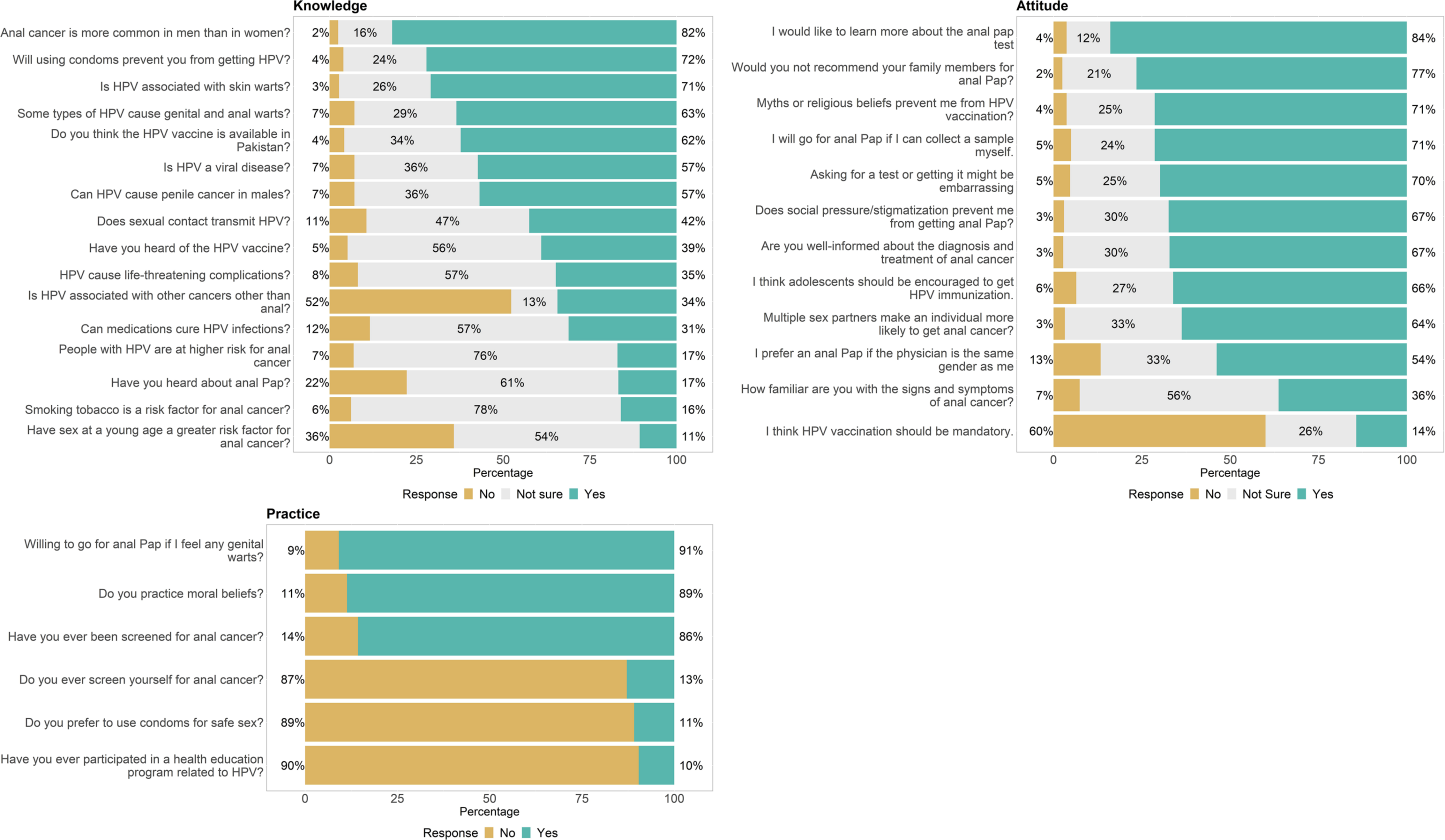
Knowledge, attitudes and practices questionnaire and responses trend in Pakistan population.

**Table 1** provides a comprehensive overview of the demographic and socio-economic characteristics of 1620 participants. Amongst, the participants were predominantly young (53.8% aged 21-30), female (61.2%), single (80.5%), unemployed (70.6%), students (67.9%), and had large families (72.3% had four or more family members). In addition, most of the participants did not disclose their income (59.5%), had a university education (66.9%), were Muslim (99.3%), and lived in KPK province (59.8%). Moreover, we evaluated KAP parameters using different questionnaires that clearly demonstrate our findings, as shown in **Figure 2**.

### 3.2 Knowledge of prevention and transmission of HPV and anal cancer

The respondents had varying levels of knowledge about HPV and anal cancer, as shown in **Table 2**. Most of the respondents (82%) knew that anal cancer is more common in men than in women and that HPV is a viral disease (57%) and is associated with other cancers other than anal (34%). However, only 39% had heard of the HPV vaccine, and only 57% knew that HPV can cause penile cancer in males. Moreover, 34% were unsure if the HPV vaccine is available in Pakistan. The respondents also had gaps in their knowledge about the transmission and prevention of HPV, as 11% did not know that sexual contact transmits HPV, 24% did not know that using condoms can prevent HPV, and 26% did not know that HPV is associated with skin warts. The respondents also had mixed knowledge about the risk factors and complications of HPV and anal cancer, as 63% knew that some types of HPV cause genital and anal warts, but only 16% knew that smoking tobacco is a risk factor for anal cancer. Similarly, 35% knew that HPV can cause life-threatening complications, but only 17% knew that people with HPV are at higher risk for anal cancer. The respondents also had limited knowledge about the screening and treatment of HPV and anal cancer, as only 17% had heard about anal Pap, and only 31% knew that medications could not cure HPV infections.

### 3.3 Attitude towards stigma and embarrassment of anal cancer screening

The survey results revealed the respondents’ attitudes towards HPV and anal cancer, as shown in **Table 3**. The majority of the respondents (67%) reported being well-informed about the diagnosis and treatment of anal cancer, and 36% were familiar with the signs and symptoms of anal cancer. However, the survey also revealed a high level of stigma and embarrassment associated with anal cancer screening, as 70% agreed that asking for a test or getting it might be embarrassing, and 54% preferred an anal Pap if the physician is the same gender as them. The survey also showed a mixed level of acceptance and willingness to learn more about the prevention and management of HPV and anal cancer, as 66% agreed that adolescents should be encouraged to get HPV immunization, 14% agreed that HPV vaccination should be mandatory, 71% agreed that they would go for an anal Pap if they can collect a sample themselves, 84% agreed that would like to learn more about the anal pap test, and 77% disagreed that they would not recommend their family members for anal Pap. The survey also showed a high level of awareness and resilience among the respondents about the risk factors and barriers for HPV and anal cancer, as 64% agreed that multiple sex partners make an individual more likely to get anal cancer, 67% agreed that social pressure and stigmatization would prevent them from anal Pap, and 71% agreed that myths or religious beliefs would prevent them from HPV vaccination.

### 3.4 Practice of preventive and curative measures

The survey results revealed the respondents’ practices related to HPV and anal cancer, as shown in **Table 4**. Most respondents (89%) reported practicing moral beliefs, and 91% reported preferring not to use condoms for safe sex. However, the survey also revealed a low level of participation and utilization of health services and programs related to HPV and anal cancer, as only 9.6% reported ever participating in a health education program related to HPV, only 14% reported ever being screened for anal cancer, only 13% reported ever screening themselves for anal cancer, and only 9.1% reported being willing to go for an anal Pap if they feel any genital warts. These findings suggest a need for more outreach and advocacy efforts to increase the awareness and demand for health services and programs related to HPV and anal cancer and to overcome the barriers and misconceptions that may prevent the general population from seeking timely and appropriate care.

### 3.5 Binary logistic regression

We used binary logistic regression analysis to explore the association between the KAP of anal cancer and various socio-demographic factors. The dependent variables were knowledge, attitude, and practice grading, while the independent variables were all collected socio-demographic variables. However, odds ratios and the 95% confidence intervals indicate the direction and magnitude of the association between each predictor and the practice grading.

#### 3.5.1 Knowledge

The dependent variable was the knowledge grading, categorized as knowledgeable (75.23%) or not-knowledgeable (24.77%) based on the scores obtained from the knowledge questions. As shown in **Table 5**, the independent variables were the socio-demographic variables. Age, marital status, occupation, family members, income, education, attitude, and practice significantly influenced the knowledge grading (p< 0.05). In addition, education is marginally significantly associated with knowledge grading, with higher education levels being more likely to be knowledgeable than lower education levels (OR = 1.0984, p = 0.05, 95% CI = 0.7226-1.6716). The odds of being knowledgeable are 1.0984 times higher for university graduates than for high school graduates. The participants who were more likely to be knowledgeable were those who were aged 10-20 years (OR = 0.7021, p = 0.00, 95% CI = 0.4561-1.0768), student (OR = 0.3773, p = 0.00, 95% CI = 0.1494-0.9410), living with four or more family members (OR = 1.5152, p = 0.00, 95% CI = 1.1338-2.0328), earning less than or equal to 50,000 rupees per month (OR = 2.1580, p = 0.00, 95% CI = 1.3329-3.5188) and having a university education (OR = 1.0984, p = 0.05, 95% CI = 0.7226-1.6716). On the other hand, the participants who were less likely to be knowledgeable were those who were older than 20 years (ORs ranging from 0.5851 to 0.0000, p = 0.00, 95% CIs ranging from 0.3010 to 2.0092), teacher (OR = 0.3738, p = 0.00, 95% CI = 0.1612-0.8568), living with two or fewer family members (OR = 1.1422, p = 0.00, 95% CI = 0.6545-1.9923), earning more than or equal to 51,000 rupees per month (ORs ranging from 0.9723 to 1.0477, p = 0.00, 95% CIs ranging from 0.6164-1.5351 to 0.7051-1.5622) and having a primary school education (OR = 0.8201, p = 0.46, 95% CI = 0.2424-2.5817).

#### 3.5.2 Attitude

The dependent variable was attitude grading, categorized as negative (21.55%) or positive (78.45%) based on the scores obtained from the attitude questions. Overall, age, gender, employment status, occupation, family members, income, resident province, practice, and knowledge were significant predictors of the attitude grading (p< 0.05). As shown in the **Table 6**, the participants who were more likely to have a positive attitude towards anal cancer prevention and screening were those who were aged 21-30 years (OR = 0.2129, p< 0.001, 95% CI = 0.1152-0.3885), female (OR = 0.7623, p< 0.001, 95% CI = 0.5367-1.0837), single (OR = 1.3786, p = 0.045, 95% CI = 0.2779-5.9927), unemployed (OR = 1.6644, p = 0.005, 95% CI = 0.8685-3.1235), student (OR = 2.8186, p< 0.001, 95% CI = 0.9474-8.0406), living with four or more family members (OR = 1.7157, p< 0.001, 95% CI = 1.0889-2.6860), earning more than 80,000 rupees per month (OR = 6.6052, p< 0.001, 95% CI = 3.1617-14.4197) and residing in Islamabad Capital Territory (OR = 1.0115, p = 0.028, 95% CI = 0.4274-2.4381). On the other hand, the participants who were less likely to have a positive attitude were those who were older than 30 years (ORs ranging from 0.0589 to 0.1781, p< 0.001, 95% CIs ranging from 0.0102-0.4461), teacher (OR = 1.2271, p< 0.001, 95% CI = 0.4603-3.1115), living with two or fewer family members (OR = 0.8782, p< 0.001, 95% CI = 0.4451-1.7421), earning less than or equal to 50,000 rupees per month (OR = 0.8279, p< 0.001, 95% CI = 0.4645-1.4647) and having a primary school education (OR = 0.2562, p< 0.001, 95% CI = 0.0623-1.2494). In addition, we did not find any significant association between attitude grading and religion (p = 0.51).

#### 3.5.3 Practices

The dependent variable was the practice grading, categorized as good (9.16%) or poor (90.84%) based on the scores obtained from the practice questions. Overall, age, gender, employment status, occupation, family members, income, resident province, knowledge, and attitude were significant predictors of the practice grading (p< 0.05). As shown in **Table 7**, the participants who were more likely to have good practice for HPV prevention were those who were aged 31-40 years (OR = 0.1323, p< 0.001, 95% CI = 0.0415-0.4081), male (OR = 0.3593, p< 0.001, 95% CI = 0.2374-0.5394), employed (OR = 3.8431, p< 0.001, 95% CI = 2.0515-7.1113), teacher (OR = 2.1890, p< 0.001, 95% CI = 0.5111-8.1261), living with four or more family members (OR = 0.2284, p< 0.001, 95% CI = 0.1126-0.4260), earning less than or equal to 50,000 rupees per month (OR = 0.1820, p< 0.001, 95% CI = 0.0905-0.3498) and residing in Islamabad Capital Territory (OR = 3.5210, p = 0.039, 95% CI = 1.1490-13.3843). On the other hand, the participants who were less likely to have a good practice were those who were older than 40 years, female, unemployed, student (OR = 0.3381, p< 0.001, 95% CI = 0.0795-1.1964), living with two or fewer family members (OR = 0.8186, p< 0.001, 95% CI = 0.2422-3.3357), earning more than 80,000 rupees per month (OR = 0.6174, 95% CI = 0.2910-1.2801) and residing in Punjab. We did not find any significant association between practice grading, marital status (p = 0.65), and religion (p = 0.54).

### 3.6 Demographic and socio-economic correlates for anal cancer KAP

**Figure 3:**
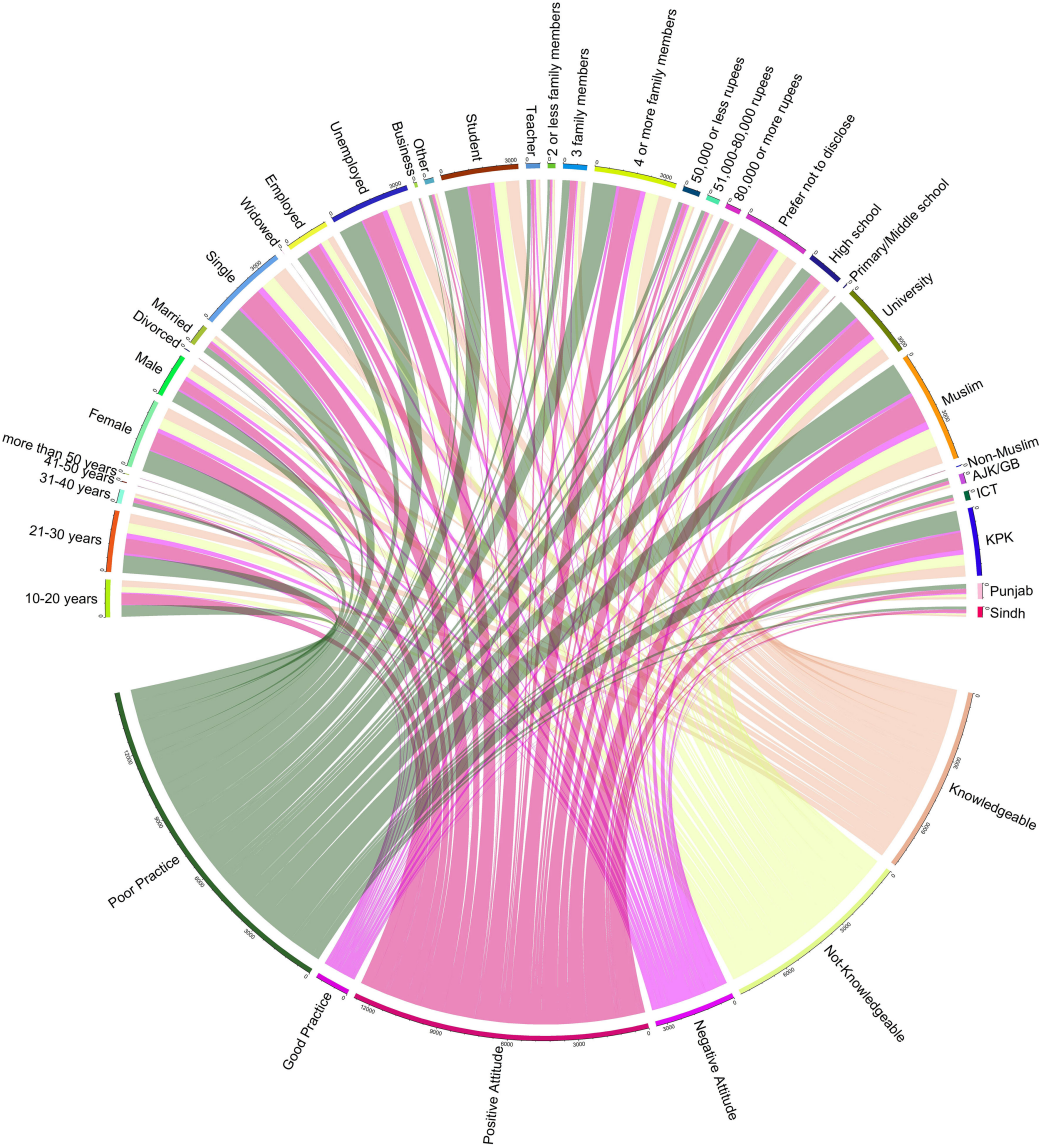
Overall distribution of Knowledge, attitudes and practices in different socio-demographic variables.

**Figure 3** shows how different demographic and socio-economic factors affected the knowledge, attitude, and Practice of HPV in Pakistan. The sample was grouped by age: 10-20 years (n=532, 33%), 21-30 years (n=872, 54%), 31-40 years (n=196, 12%), and 41-50 years (n=12, 0.75%). The 10-20 years group had the highest percentage of knowledgeable (53%, n=284), positive (90%, n=480), and good practicing (4.5%, n=24) respondents. The 21-30 years group had the second highest percentage of knowledgeable (48%, n=416), positive (73%, n=640), and good practicing (11%, n=96) respondents. The 31-40 years group had the third highest percentage of knowledgeable (45%, n=88), positive (75%, n=148), and good practicing (14%, n=28) respondents. The 41-50 years group had the lowest percentage of knowledgeable (33%, n=4), positive (33%, n=4), and good practicing (0%) respondents.

Moreover, females were more knowledgeable (n=491, 30.6%), positive (n=814, 50.9%), and good practicing (n=56, 3.5%) than males (n=309, 19.3%; n=458, 28.6%; n=92, 5.7%). Single and widowed respondents were more knowledgeable (n=656, 40.9%; n=0, 0%), positive (n=1040, 64.8%; n=4, 0.25%) and good practicing (n=120, 7.5%; n=0, 0%) than married and divorced respondents (n=140, 8.7%; n=4, 0.25%; n=220, 13.7%; n=8, 0.5%; n=28, 1.7%; n =0,0%). Business owners and teachers were more knowledgeable (n =68,4.2%; n =40, 2.5%), positive (n =128, 8%; n =64,4%) and good practicing (n =12, 0.75%; n =4, 0.25%) than other occupations (n =96, 6%; n =584, 36%; n =28, 1.7%; n =24, 1.5%; n =176,11%; n =920, 57%; n =56, 3.5%; n =56, 3.5%; n =8, 0.5%; n =76, 4.7%; n =8, 0.5%; n =8, 0.5%). Respondents from ICT and Punjab were more knowledgeable (n =232, 14%; n =112, 7%), positive (n =376, 23%; n =80, 5%) and good practicing (n =16, 1%; n =8, 0.5%) than other regions (n =32, 2%; n =48, 3%; n =16, 1%; n =56, 3.5%; n =56, 3.5%; n =32, 2%; n =8, 0.5%; n =8, 0.5%; n =4, 0.25%).

## 4 Discussion

The survey participants were 1620 individuals who answered questions about their knowledge, attitude, and practices regarding anal cancer and HPV infection in Pakistan. Most of the participants were young, female, single, unemployed, students, and had large families. Most of them did not disclose their income, had a university education, were Muslim, and lived in KPK province. Our results show that the respondents had varying knowledge about anal cancer and HPV infection but low awareness about the HPV vaccine and its availability in Pakistan. The respondents also had mixed attitudes toward HPV prevention, screening, and treatment. For example, 82% of the respondents knew that anal cancer is more common in men than in women, but only 39% had heard of the HPV vaccine, and only 57% knew that it could prevent penile cancer in males.

Similarly, 17% of the respondents had heard of anal Pap screening, but only 31% knew medications could not cure HPV infections. These findings are consistent with some published studies on HPV KAP in Pakistan and other countries. For example, a study by Ejaz et al. (33) found that none of the MSM and transgender women participants had heard about HPV prevention, including vaccination and anal Pap screening, and lacked knowledge of HPV and its health consequences. The participants expressed a positive attitude toward HPV prevention but acknowledged that services for sexually transmitted infections (STI) were inadequate and not prioritized by the government or the local community-based organizations (CBOs). Another study by Al Yahyai et al. (34) found that 67.5% and 50.9% had heard of cervical cancer and Pap smear testing, respectively; however, only 13.4% and 10.9% demonstrated high levels of knowledge concerning these topics. In addition, knowledge was significantly correlated with educational level, type of educational qualification (i.e., whether the degree was in healthcare), monthly income, and employment status (p-value 0.05 for each). Only 15.7% of the respondents had undertaken a Pap smear examination in the past, but 42.7% were willing to do so in the future.

Furthermore, a study by Husnain et al. (35) found that the level of awareness of HPV infection and vaccine among men and women in Bahrain was low, with only 13.5% of the respondents having heard of HPV and more than 90% of the participants agreed on the need for educating the community about the HPV infection. The respondents also had negative attitudes towards receiving the vaccine, mainly due to fear of side effects, lack of trust in its efficacy, and religious or cultural reasons. In contrast, a study of Indonesian urban citizens reported poor knowledge but good attitude toward HPV infection, cervical cancer, and HPV vaccination. Overall, in the general population, men and women respectively, 50.8%, 32.4%, and 53.6% had good knowledge, but 82% expressed a positive attitude towards receiving the vaccine if offered for free or at low cost. The main predictors for good knowledge were female sex, higher education, and higher mother’s education. However, their knowledge was weakly correlated towards attitude (ρ = 0.385) but moderately correlated with practice (ρ = 0.485); attitude was moderately correlated with practice (ρ = 0.577) (26). Collectively, these studies suggest variations in the KAP of anal cancer and a basic understanding of HPV among different population groups and regions, depending on the level of education, income, age, gender, and cultural factors.

Current study results revealed that respondents had varying awareness and opinions about HPV prevention, screening, and treatment. However, participants had a fair amount of knowledge and confidence regarding the signs, symptoms, diagnosis, and treatment of anal cancer. Most of them (67%) reported being well-informed, and 36% were aware of the signs and symptoms of anal cancer. In contrast, findings are inconsistent with previous studies that reported low levels of awareness and education about anal cancer among the general population (36, 37). Our survey also revealed a high level of stigma and embarrassment related to anal cancer screening, as 70% of the participants agreed that asking for a test or getting it might be embarrassing, and 54% preferred an anal Pap if the physician was the same gender as them. These findings align with previous studies that have identified stigma, embarrassment, and gender preference as barriers to anal cancer screening (38–40). Moreover, the survey also demonstrated a mixed level of acceptance and willingness to learn more about the prevention and management of HPV and anal cancer among the participants.

Furthermore, as many as 66% of them agreed that adolescents should be encouraged to get HPV immunization, 14% agreed that HPV vaccination should be mandatory, 71% agreed that they would go for an anal Pap if they could collect a sample themselves, 84% agreed that they would like to learn more about the anal pap test, and 77% of participants would not recommend their family members for anal Pap. These findings suggest a positive attitude and readiness for HPV vaccination for adolescents but negative for anal cancer screening. In contrast, findings were reported in previous studies that have reported high levels of acceptance and willingness for HPV vaccination (41, 42) and high hesitancy for anal screening (30, 43, 44). One possible reason for this difference is that our study population may have different levels of awareness, knowledge, or stigma about HPV infection and anal cancer screening than the previous studies. Secondly, another possible reason is that our study used a self-administered questionnaire, whereas previous studies used face-to-face interviews or clinical examinations (45–47). This may have influenced the participants’ responses, as they may have been more honest or comfortable answering sensitive questions privately.

The survey also showed a high level of awareness and resilience among the participants regarding the risk factors and barriers for HPV and anal cancer. As many as 64% agreed that multiple sex partners make an individual more likely to get anal cancer, 67% responded that social pressure and stigmatization would prevent them from anal Pap, and 71% responded that myths or religious beliefs would prevent HPV vaccination. This is consistent with previous studies that found that most men who have sex with men knew the link between HPV infection and anal cancer. Our findings are consistent with previous studies; some men who have sex with men may avoid HPV vaccination and anal cancer screening due to social pressure, stigma, myths, or religious beliefs (44, 48, 49), embarrassment (50, 51), lack of familiarity with tests (50, 52), despite knowing the link between HPV infection and anal cancer (31, 53). Herein, it is suggested that peer education, counseling, or community engagement may help overcome the barriers and increase the uptake of HPV vaccination and anal cancer screening among men who have sex with men (54, 55).

The current survey indicates a low level of participation and utilization of health services and programs related to HPV and anal cancer among the participants. Similarly, these findings are consistent with previous studies that have reported high levels of moral beliefs among the general population (56–58) and low condom usage (59–61). Similarly, a study by Grandahl et al. (62) from Thailand reported socio-demographic characteristics and religious convictions and reported links between parents’ knowledge, beliefs, and acceptance of the HPV vaccination for their young daughter. Compared to parents who said religion was less significant, parents who said religion was essential were more in favor of the HPV vaccine. Additionally, the current survey also revealed a low level of participation and utilization of health services and programs related to HPV and anal cancer among the participants. The only question that received a high percentage of positive responses (91%) was whether the participants would be willing to go for an anal Pap if they felt genital warts, suggesting a potential interest in early detection and treatment. Only 13% of them reported ever participating in a health education program related to HPV, and only 11% of them reported ever being screened for anal cancer. These findings contrast with previous studies that have reported higher levels of participation and utilization of health services and programs related to HPV and anal cancer among the general population (63–65). The current study strongly recommends that Pakistan needs more outreach and advocacy efforts to increase the awareness and demand for health services and programs related to HPV and anal cancer and to overcome the barriers and misconceptions that may prevent the general population from seeking timely and appropriate care (63, 66).

This is the first study in Pakistan to explore how young adults attending a university/college think and feel about HPV and anal cancer and what they do to prevent them. We found that males and females had similar knowledge, attitudes, and screening behaviors, but they were significantly low. Most participants did not know much about HPV and anal cancer, felt ashamed and embarrassed to talk about them, and faced many socio-cultural challenges to protect themselves. They also reported following moral beliefs and avoiding condoms for safe sex. However, they hardly ever participated in health education programs or screenings related to HPV and anal cancer. Only a few of them had ever learned about HPV, checked themselves for anal cancer, or got tested for anal cancer by a doctor. Even fewer were willing to get an anal Pap test if they noticed genital warts. These findings show an urgent need for more awareness campaigns and support services to help people understand and access health care for HPV and anal cancer and overcome the myths and barriers that may prevent them from getting timely and proper treatment. We also found that young adults in our study were not well informed or motivated to get vaccinated or screened for HPV and anal cancer, which means the existing prevention efforts have not reached them. Consequently, this shows the importance of training health professionals and educators to inform young adults about this issue.

Our study has some limitations, such as being unable to represent all young adults in Pakistan because most participants were from the KPK and Punjab provinces. This study used a self-administered questionnaire as the data collection method, which may introduce misclassification bias due to the lack of validation of the participants’ responses. The anonymity of the questionnaire may not have eliminated the social desirability bias, as the topic’s controversial nature may have influenced the participants. Moreover, we lacked detailed data on their sexual behavior, HPV vaccination status, and anal cancer screening history, which could confound their disease risk. However, our study is among the pioneer studies to assess HPV and anal cancer awareness among young adults in Pakistan. Thus, it provides valuable insights for future research studies and public health interventions to enhance HPV and anal cancer screening and prevention.

## 5 Conclusion

This study demonstrated that young adults in Pakistan have inadequate awareness, elevated stigma and embarrassment, and multiple socio-cultural challenges regarding HPV and anal cancer. These factors may impede their engagement and utilization of health services and programs that can prevent and treat these diseases. Hence, there is a need for more education and awareness campaigns and more available and affordable health services and programs to address the gaps and barriers in the prevention and management of HPV and anal cancer among this population. Furthermore, there is a need for more research and policy initiatives to comprehend and address the socio-cultural factors that influence the knowledge, attitudes, and experiences of young adults in Pakistan regarding HPV and anal cancer. By doing so, the health outcomes and quality of life of this population can be enhanced.

## Data availability statement

The original contributions presented in the study are included in the article/supplementary material. Further inquiries can be directed to the corresponding authors.

## Ethics statement

Ethical approval was obtained from the Departmental Bioethical Committee of The University of Haripur (UOH-RC-EA-142). Informed consent was taken from all participants before they participated in the study. In the case of participants under 18 years old, the participant’s legal guardian provided written informed consent to participate in this study.

## Author contributions

UA: Conceptualization, Data curation, Methodology, Writing – original draft, Writing – review & editing. WN: Conceptualization, Data curation, Methodology, Writing – original draft. AK: Data curation, Investigation, Methodology, Writing – review & editing. TM: Formal Analysis, Methodology, Software, Validation, Visualization, Writing – original draft. ShK: Conceptualization, Data curation, Methodology, Writing – original draft. SuK: Data curation, Investigation, Methodology, Project administration, Formal Analysis, Writing – review & editing. XG: Conceptualization, Formal Analysis, Project administration, Supervision, Writing – review & editing. ZY: Funding acquisition, Project administration, Supervision, Writing – review & editing. JL: Funding acquisition, Project administration, Resources, Supervision, Writing – review & editing. AN: Writing – review & editing.

## References

[1] Gottlieb SL Low N Newman LM Bolan G Kamb M Broutet N. Toward global prevention of sexually transmitted infections (STIs): the need for STI vaccines. Vaccine (2014) 32:1527–35. doi: 10.1016/j.vaccine.2013.07.087 PMC6794147 24581979

[2] Martinelli M Musumeci R Sechi I Sotgiu G Piana A Perdoni F. Prevalence of human papillomavirus (HPV) and other sexually transmitted infections (STIs) among Italian women referred for a colposcopy. Int J Environ Res Public Health (2019) 16:5000. doi: 10.3390/ijerph16245000 31818033 PMC6950209

[3] Chaturvedi AK. Beyond cervical cancer: burden of other HPV-related cancers among men and women. J Adolesc Health (2010) 46:S20–6. doi: 10.1016/j.jadohealth.2010.01.016 20307840

[4] Araldi RP Sant’Ana TA Módolo DG de Melo TC Spadacci-Morena DD de Cassia Stocco R. The human papillomavirus (HPV)-related cancer biology: An overview. Biomed pharmacother (2018) 106:1537–56. doi: 10.1016/j.biopha.2018.06.149 30119229

[5] Chan PK Chang AR Cheung JL Chan DP Xu L Tang NL. Determinants of cervical human papillomavirus infection: differences between high-and low-oncogenic risk types. J Infect diseases (2002) 185:28–35. doi: 10.1086/338010 11756978

[6] Awan UA Khattak AA Ahmed N Guo X Akhtar S Kamran S. An updated systemic review and meta-analysis on human papillomavirus in breast carcinogenesis. Front Oncol (2023) 13:1219161. doi: 10.3389/fonc.2023.1219161 37711194 PMC10498127

[7] Wei F Gaisa MM D'Souza G Xia N Giuliano AR Hawes SE. Epidemiology of anal human papillomavirus infection and high-grade squamous intraepithelial lesions in 29 900 men according to HIV status, sexuality, and age: a collaborative pooled analysis of 64 studies. Lancet HIV (2021) 8:e531–43. doi: 10.1016/S2352-3018(21)00108-9 PMC8408042 34339628

[8] Zhao X-L Hu S-Y Zhang Q Dong L Feng R-M Han R. High-risk human papillomavirus genotype distribution and attribution to cervical cancer and precancerous lesions in a rural Chinese population. J gynecologic Oncol (2017) 28:30. doi: 10.3802/jgo.2017.28.e30 PMC5447139 28541628

[9] Lin C Franceschi S Clifford GM. Human papillomavirus types from infection to cancer in the anus, according to sex and HIV status: a systematic review and meta-analysis. Lancet Infect Diseases (2018) 18:198–206. doi: 10.1016/S1473-3099(17)30653-9 29158102 PMC5805865

[10] Palefsky JM Lee JY Jay N Goldstone SE Darragh TM Dunlevy HA. Treatment of anal high-grade squamous intraepithelial lesions to prevent anal cancer. New Engl J Med (2022) 386:2273–82. doi: 10.1056/NEJMoa2201048 PMC9717677 35704479

[11] Khan S Jaffer NN Khan MN Rai MA Shafiq M Ali A. Human papillomavirus subtype 16 is common in Pakistani women with cervical carcinoma. Int J Infect diseases (2007) 11:313–7. doi: 10.1016/j.ijid.2006.06.007 17291804

[12] Yanofsky VR Patel RV Goldenberg G. Genital warts: a comprehensive review. J Clin aesthetic Dermatol (2012) 5:25. PMC3390234 22768354

[13] De Martel C Plummer M Vignat J Franceschi S. Worldwide burden of cancer attributable to HPV by site, country and HPV type. Int J cancer (2017) 141:664–70. doi: 10.1002/ijc.30716 PMC5520228 28369882

[14] Deshmukh AA Suk R Shiels MS Sonawane K Nyitray AG Liu Y. Recent trends in squamous cell carcinoma of the anus incidence and mortality in the United States, 2001–2015. JNCI: J Natl Cancer Institute. (2020) 112:829–38. doi: 10.1093/jnci/djz219 PMC7825484 31742639

[15] Albuquerque A Nathan M Cappello C Dinis-Ribeiro M. Anal cancer and precancerous lesions: a call for improvement. Lancet Gastroenterol Hepatology (2021) 6:327–34. doi: 10.1016/S2468-1253(20)30304-6 33714370

[16] Idrees R Fatima S Abdul-Ghafar J Raheem A Ahmad Z. Cancer prevalence in Pakistan: meta-analysis of various published studies to determine variation in cancer figures resulting from marked population heterogeneity in different parts of the country. World J Surg Oncol (2018) 16:1–11. doi: 10.1186/s12957-018-1429-z 29976196 PMC6034324

[17] Cancer IIICoHa. Human Papillomavirus and Related Diseases Report-Pakistan (2023). Available at: https://hpvcentre.net/statistics/reports/PAK.pdf.

[18] Shafiq M Ali SH. Sexually transmitted infections in Pakistan. Lancet Infect Diseases (2006) 6:321–2. doi: 10.1016/S1473-3099(06)70474-1 16728317

[19] Rajabali A Khan S Warraich HJ Khanani MR Ali SH. HIV and homosexuality in Pakistan. Lancet Infect diseases (2008) 8:511–5. doi: 10.1016/S1473-3099(08)70183-X 18652997

[20] Samarasekera U. Pakistan's growing HIV epidemic. Lancet (2022) 400:2031. doi: 10.1016/S0140-6736(22)02530-2 36502834

[21] Faisel A Cleland J. Migrant men: a priority for HIV control in Pakistan? Sexually transmitted infections (2006) 82:307–10. doi: 10.1136/sti.2005.018762 PMC2564715 16877580

[22] Ahmed MA Zafar T Brahmbhatt H Imam G Hassan SU Bareta JC. HIV/AIDS risk behaviors and correlates of injection drug use among drug users in Pakistan. J Urban Health (2003) 80:321–9. doi: 10.1093/jurban/jtg034 PMC3456274 12791807

[23] Haque N Zafar T Brahmbhatt H Imam G Ul Hassan S Strathdee S. High-risk sexual behaviours among drug users in Pakistan: implications for prevention of STDs and HIV/AIDS. Int J STD AIDS (2004) 15:601–7. doi: 10.1258/0956462041724172 15339368

[24] Kuo I Galai N Thomas DL Zafar T Ahmed MA Strathdee SA. High HCV seroprevalence and HIV drug use risk behaviors among injection drug users in Pakistan. Harm Reduction J (2006) 3:1–10. doi: 10.1186/1477-7517-3-26 PMC1564387 16914042

[25] Cinar İO Ozkan S Aslan GK Alatas E. Knowledge and behavior of university students toward human papillomavirus and vaccination. Asia-Pacific J Oncol Nursing (2019) 6:300–7. doi: 10.4103/apjon.apjon_10_19 PMC6518985 31259227

[26] Winarto H Habiburrahman M Dorothea M Wijaya A Nuryanto KH Kusuma F. Knowledge, attitudes, and practices among Indonesian urban communities regarding HPV infection, cervical cancer, and HPV vaccination. PloS One (2022) 17:e0266139. doi: 10.1371/journal.pone.0266139 35552546 PMC9098048

[27] Leung JTC Law C-k. Revisiting knowledge, attitudes and practice (KAP) on human papillomavirus (HPV) vaccination among female university students in Hong Kong. Hum Vaccines immunotherapeutics (2018) 14:924–30. doi: 10.1080/21645515.2017.1415685 PMC5893186 29232166

[28] Ortiz AP García-Camacho SI Ramos-Cartagena JM Colón-López V Estremera-Rodríguez LM Berríos-Toledo KM. Knowledge, attitudes, and experiences of anal cancer and anal cancer screening among a clinical sample of Hispanic women. J lower genital tract disease (2021) 25:98. doi: 10.1097/LGT.0000000000000598 PMC8171435 33660677

[29] Ong JJ Chen M Grulich A Walker S Temple-Smith M Bradshaw C. Exposing the gaps in awareness, knowledge and estimation of risk for anal cancer in men who have sex with men living with HIV: a cross-sectional survey in Australia. J Int AIDS Society (2015) 18:19895. doi: 10.7448/IAS.18.1.19895 PMC4380906 25828269

[30] Rodriguez SA Higashi RT Betts AC Ortiz C Tiro JA Luque AE. Anal cancer and anal cancer screening knowledge, attitudes, and perceived risk among women living with HIV. J lower genital tract disease (2021) 25:43. doi: 10.1097/LGT.0000000000000578 PMC7750264 33149011

[31] Wells JS Flowers L Paul S Nguyen ML Sharma A Holstad M. Knowledge of anal cancer, anal cancer screening, and HPV in HIV-positive and high-risk HIV-negative women. J Cancer Education (2020) 35:606–15. doi: 10.1007/s13187-019-01503-8 PMC6732243 30850945

[32] Guttman L. A basis for analyzing test-retest reliability. Psychometrika (1945) 10:255–82. doi: 10.1007/BF02288892 21007983

[33] Ejaz M Ekström AM Ahmed A Haroon A Ali D Ali TS. Human Papillomavirus associated prevention: Knowledge, attitudes, and perceived risks among men who have sex with men and transgender women in Pakistan: A qualitative study. BMC Public Health (2022) 22:1–12. doi: 10.1186/s12889-022-12775-z 35193544 PMC8864907

[34] Al Yahyai T Al Raisi M Al Kindi R. Knowledge, attitudes, and practices regarding cervical cancer screening among Omani women attending primary healthcare centers in Oman: a cross-sectional survey. Asian Pacific J Cancer Prevention: APJCP (2021) 22:775. doi: 10.31557/APJCP.2021.22.3.775 PMC8286659 33773541

[35] Husain Y Alalwan A Al-Musawi Z Abdulla G Hasan K Jassim G. Knowledge towards human papilloma virus (HPV) infection and attitude towards its vaccine in the Kingdom of Bahrain: cross-sectional study. BMJ Open (2019) 9:e031017. doi: 10.1136/bmjopen-2019-031017 PMC6773289 31562156

[36] Waller J McCaffery K Forrest S Szarewski A Cadman L Wardle J. Awareness of human papillomavirus among women attending a well woman clinic. Sexually Transmitted Infections (2003) 79:320–2. doi: 10.1136/sti.79.4.320 PMC1744711 12902585

[37] Tfaily MA Naamani D Kassir A Sleiman S Ouattara M Moacdieh MP. Awareness of colorectal cancer and attitudes towards its screening guidelines in Lebanon. Ann Global Health (2019) 85:75. doi: 10.5334/aogh.2437 PMC6634322 31148437

[38] Koskan AM Fernandez-Pineda M. Anal cancer prevention perspectives among foreign-born latino HIV-infected gay and bisexual men. Cancer Control (2018) 25:1073274818780368. doi: 10.1177/1073274818780368 29925247 PMC6028166

[39] Consedine NS Reddig MK Ladwig I Broadbent EA. Gender and ethnic differences in colorectal cancer screening embarrassment and physician gender preferences. Oncol Nurs Forum (2011) 38(6):409–17. doi: 10.1188/11.ONF.E409-E417 22037340

[40] Ali MA Ahmad F Morrow M. Somali’s perceptions, beliefs and barriers toward breast, cervical and colorectal cancer screening: a socioecological scoping review. Int J Migration Health Soc Care (2021) 17:224–38. doi: 10.1108/IJMHSC-06-2020-0059

[41] Reiter PL Brewer NT McRee A-L Gilbert P Smith JS. Acceptability of HPV vaccine among a national sample of gay and bisexual men. Sexually transmitted diseases (2010) 37:197. doi: 10.1097/OLQ.0b013e3181bf542c 20118831 PMC4018212

[42] Maharajan MK Rajiah K Num KSF Yong NJ. Knowledge of human papillomavirus infection, cervical cancer and willingness to pay for cervical cancer vaccination among ethnically diverse medical students in Malaysia. Asian Pacific J Cancer Prev (2015) 16:5733–9. doi: 10.7314/APJCP.2015.16.14.5733 26320444

[43] Grace D Gaspar M Paquette R Rosenes R Burchell AN Grennan T. HIV-positive gay men’s knowledge and perceptions of Human Papillomavirus (HPV) and HPV vaccination: A qualitative study. PloS One (2018) 13:e0207953. doi: 10.1371/journal.pone.0207953 30496221 PMC6264470

[44] Newman PA Roberts KJ Masongsong E Wiley D. Anal cancer screening: barriers and facilitators among ethnically diverse gay, bisexual, transgender, and other men who have sex with men. J gay lesbian Soc services (2008) 20:328–53. doi: 10.1080/10538720802310733 PMC3002049 21165164

[45] Russo S Mccaffery K Ellard J Poynten M Prestage G Templeton D. Experience and psychological impact of anal cancer screening in gay, bisexual and other men who have sex with men: a qualitative study. Psycho-Oncology (2018) 27:125–31. doi: 10.1002/pon.4480 28635044

[46] Donà MG Benevolo M Vocaturo A Palamara G Latini A Giglio A. Anal cytological abnormalities and epidemiological correlates among men who have sex with men at risk for HIV-1 infection. BMC cancer (2012) 12:1–8. doi: 10.1186/1471-2407-12-476 23072547 PMC3517502

[47] Liu X Lin H Chen X Shen W Ye X Lin Y. Prevalence and genotypes of anal human papillomavirus infection among HIV-positive vs. HIV-negative men Taizhou China. Epidemiol Infection (2019) 147:e117. doi: 10.1017/S0950268818003205 PMC6518779 30868975

[48] Wong LP Wong P-F Megat Hashim MMAA Han L Lin Y Hu Z. Multidimensional social and cultural norms influencing HPV vaccine hesitancy in Asia. Hum Vaccines immunotherapeutics (2020) 16:1611–22. doi: 10.1080/21645515.2020.1756670 PMC7482900 32429731

[49] Hendry M Lewis R Clements A Damery S Wilkinson C. “HPV? Never heard of it!”: a systematic review of girls’ and parents’ information needs, views and preferences about human papillomavirus vaccination. Vaccine (2013) 31:5152–67. doi: 10.1016/j.vaccine.2013.08.091 24029117

[50] Blankenship SA Debnath P Szlachta-McGinn AW Maguire K Garcia JJ Aserlind A. Knowledge and acceptability of anal cytology screening among women. J lower genital tract disease (2016) 20:90–6. doi: 10.1097/LGT.0000000000000151 26461234

[51] Ferris D Lambert R Waller J Dickens P Kabaria R Han C-S. Women’s knowledge and attitudes toward anal Pap testing. J lower genital tract disease (2013) 17:463–8. doi: 10.1097/LGT.0b013e3182760ad5 23774075

[52] Battaglia TA Gunn CM McCoy ME Mu HH Baranoski AS Chiao EY. Beliefs about anal cancer among HIV-infected women: barriers and motivators to participation in research. Women's Health Issues (2015) 25:720–6. doi: 10.1016/j.whi.2015.06.008 PMC4641840 26253825

[53] Pitts MK Fox C Willis J Anderson J. What do gay men know about human papillomavirus? Australian gay men's knowledge and experience of anal cancer screening and human papillomavirus. Sexually transmitted diseases (2007) 34:170–3. doi: 10.1097/01.olq.0000230436.83029.ce 16837830

[54] Vorsters A Arbyn M Baay M Bosch X de Sanjosé S Hanley S. Overcoming barriers in HPV vaccination and screening programs. Papillomavirus Res (2017) 4:45–53. doi: 10.1016/j.pvr.2017.07.001 29179869 PMC7268103

[55] Cartmell KB Mzik CR Sundstrom BL Luque JS White A Young-Pierce J. HPV vaccination communication messages, messengers, and messaging strategies. J Cancer Education (2019) 34:1014–23. doi: 10.1007/s13187-018-1405-x PMC9673969 30054900

[56] Grandahl M Tydén T Westerling R Nevéus T Rosenblad A Hedin E. To consent or decline HPV vaccination: a pilot study at the start of the national school-based vaccination program in Sweden. J school Health (2017) 87:62–70. doi: 10.1111/josh.12470 27917484 PMC5157750

[57] Das A Madhwapathi V Davies P Brown G Dearnley E Spencer A. Knowledge and acceptability of the HPV vaccine by school children and their parents in Birmingham. Vaccine (2010) 28:1440–6. doi: 10.1016/j.vaccine.2009.11.041 20005317

[58] Awan UA Guo X Khattak AA Hassan U Bashir S. HPV vaccination and cervical cancer screening in Afghanistan threatened. Lancet Infect Dis (2023) 23(2):141–2. doi: 10.1016/S1473-3099(22)00868-4 36610440

[59] Izudi J Okello G Semakula D Bajunirwe F. Low condom use at the last sexual intercourse among university students in sub-Saharan Africa: Evidence from a systematic review and meta-analysis. PloS One (2022) 17:e0272692. doi: 10.1371/journal.pone.0272692 35947583 PMC9365151

[60] Deering KN Bhattacharjee P Bradley J Moses SS Shannon K Shaw SY. Condom use within non-commercial partnerships of female sex workers in southern India. BMC Public Health (2011) 11:1–12. doi: 10.1186/1471-2458-11-S6-S11 22376171 PMC3287549

[61] Tschann JM Flores E De Groat CL Deardorff J Wibbelsman CJ. Condom negotiation strategies and actual condom use among Latino youth. J Adolesc Health (2010) 47:254–62. doi: 10.1016/j.jadohealth.2010.01.018 PMC2923590 20708564

[62] Grandahl M Chun Paek S Grisurapong S Sherer P Tyden T Lundberg P. Parents’ knowledge, beliefs, and acceptance of the HPV vaccination in relation to their socio-demographics and religious beliefs: A cross-sectional study in Thailand. PloS One (2018) 13:e0193054. doi: 10.1371/journal.pone.0193054 29447271 PMC5814087

[63] Ejaz M Ekström AM Ali TS Salazar M Ahmed A Ali D. Integration of human papillomavirus associated anal cancer screening into HIV care and treatment program in Pakistan: perceptions of policymakers, managers, and care providers. BMC Public Health (2023) 23:1–12. doi: 10.1186/s12889-023-15896-1 37259085 PMC10230466

[64] Markowitz LE Dunne EF Saraiya M Chesson HW Curtis CR Gee J. Human papillomavirus vaccination: recommendations of the Advisory Committee on Immunization Practices (ACIP). Morbidity Mortality Weekly Report: Recommendations Rep (2014) 63:1–30. 25167164

[65] Ladner J Besson M-H Hampshire R Tapert L Chirenje M Saba J. Assessment of eight HPV vaccination programs implemented in lowest income countries. BMC Public Health (2012) 12:1–8. doi: 10.1186/1471-2458-12-370 22621342 PMC3419135

[66] Awan UA Khattak AA. Has Pakistan failed to roll back HPV?. Lancet Oncol (2022) 23(5):e204. doi: 10.1016/S1470-2045(22)00141-3 35489348

